# Ocean acidification, more than warming or heatwaves, constrains shoaling behaviour in a range‐extending fish through habitat simplification

**DOI:** 10.1111/1365-2656.70270

**Published:** 2026-05-11

**Authors:** Angus Mitchell, Sean D. Connell, Mary E. Hart, Ben P. Harvey, Sylvain Agostini, Davide Spatafora, Michael Izumiyama, David J. Booth, Timothy Ravasi, Ivan Nagelkerken

**Affiliations:** ^1^ Southern Seas Ecology Laboratories, School of Biological Sciences Adelaide University Adelaide South Australia Australia; ^2^ Shimoda Marine Research Center University of Tsukuba Shimoda Shizuoka Japan; ^3^ Labex ICONA International CO_2_ Natural Analogues Network Japan; ^4^ Department of Integrative Marine Ecology Sicily, Stazione Zoologica Anton Dohrn, Lungomare Cristoforo Colombo (Complesso Roosevelt) Palermo Italy; ^5^ Marine Climate Change Unit Okinawa Institute of Science and Technology (OIST) Okinawa Japan; ^6^ School of the Life Sciences University of Technology Sydney Ultimo New South Wales Australia; ^7^ Present address: School of the Life Sciences University of Technology Sydney Ultimo New South Wales Australia; ^8^ Present address: UMR Entropie, IRD, IFREMER, Universite de la Reunion, Universite de Nouvelle Caledonie Noumea New Caledonia

**Keywords:** behavioural plasticity, climate change, collective behaviour, marine heatwaves, natural analogues, ocean acidification, ocean warming, reef fish

## Abstract

1. Social context is a critical yet underexplored determinant of behavioural resilience to climate change. Group living can buffer individuals against environmental stress through enhanced vigilance, reduced predation risk and improved foraging efficiency.
2. However, whether these behavioural expressions persist under chronic (warming, acidification) and acute (marine heatwaves) climate stressors remains unclear. Using natural climate analogues spanning present‐day, ocean warming and combined warming–acidification reefs, we quantified how shoal size influences behavioural expression in a range‐extending reef fish (*Pomacentrus coelestis*).
3. Across all climate conditions, fish in larger shoals consistently exhibited higher foraging and activity levels and reduced risk‐avoidance behaviours, whereas direct effects of warming, acidification and heatwaves on behaviour were negligible.
4. In contrast, ocean acidification most likely constrained collective behaviour indirectly by simplifying benthic habitats, where fish densities were 84% lower than at the warming reef, resulting in shoals that were up to 79% smaller than the Warming and Control reefs.
5. Combined, our data suggest that shoal size mediates behavioural expression between foraging and predator avoidance and that acidification‐driven habitat simplification can alter behavioural expression indirectly by reducing fish densities and the formation of large shoals.
6. We conclude that climate change can indirectly modify behavioural expression in shoal‐forming fishes through habitat‐driven erosion of social structure.

## Graphical abstract

We show that ocean acidification, more than warming or marine heatwaves, alters shoaling behaviour indirectly by simplifying reef habitat and reducing population densities and shoal sizes. Because fish behaviour can be strongly mediated by shoal size, climate‐driven habitat change may reshape social dynamics that influence how range‐extending fishes function at their leading range edges.

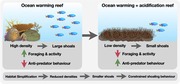

## 1 INTRODUCTION

Climate change is transforming ecosystems across the globe, altering biodiversity, community composition and the functioning of ecological networks (Pecl et al., 2017; Pinsky et al., 2020). Rising temperatures, shifting precipitation patterns and increasing frequency and magnitude of climatic extremes are reshaping terrestrial and aquatic systems alike, often pushing ecosystems towards thresholds of irreversible change (Scheffers et al., 2016; Smale et al., 2019). Marine ecosystems are particularly vulnerable compared to terrestrial systems because ocean warming, acidification and deoxygenation act at broad spatial scales, occur rapidly and directly alter the physical and chemical properties of seawater, leaving many marine organisms with limited capacity to avoid or buffer exposure (Burrows et al., 2011; Pörtner et al., 2014). The oceans have absorbed more than 90% of excess atmospheric heat and nearly 30% of all anthropogenic CO_2_ emissions, driving long‐term warming, acidification and deoxygenation, while also intensifying acute extreme events such as marine heatwaves (Frölicher & Laufkötter, 2018; Smale et al., 2019). These changes are pronounced in ocean warming hotspots, where rapid environmental change and recurrent extremes are accelerating ecological transitions (Pecl et al., 2017; Smale et al., 2019). Marine taxa are already responding by shifting poleward or into deeper habitats at rates far exceeding those of terrestrial organisms (Pecl et al., 2017), creating novel assemblages and altering long‐standing biogeographic boundaries. Such redistributions, combined with changes in demographic performance and life‐history traits, are reorganising communities, disrupting interaction networks and eroding ecosystem resilience (Nagelkerken & Connell, 2015; Pinsky et al., 2020). Ultimately, species' persistence under climate change will hinge not only on physiological limits but also on the capacity for behavioural plasticity and ecological interactions to buffer species against rapid and extreme environmental change.

Behavioural plasticity has been proposed as a key mechanism by which animals may adapt to both chronic (ocean warming and acidification) and acute (e.g. heatwaves) climate stressors (Donelson et al., 2019; Fox et al., 2019). Behavioural plasticity allows individuals to adjust activity patterns, resource use and social interactions in real time, potentially buffering fitness against novel or stressful conditions (Tuomainen & Candolin, 2011; Wong & Candolin, 2015). In fishes, behaviours such as foraging, anti‐predator responses and habitat use are highly plastic, and these shifts can influence energy balance, survival and ecological interactions (Nagelkerken et al., 2023). However, plasticity may be constrained when multiple climate stressors act simultaneously, as ocean warming, acidification and heatwaves can interact to disrupt physiological performance, habitat structure and ecological communities (Harvey et al., 2022). Understanding whether behavioural plasticity enables organisms to buffer against these combined stressors is therefore central to predicting resilience under climate change.

A central but underexplored aspect of behavioural plasticity is the role of social context, particularly group size. Group living is a hallmark of many animals, providing ecological benefits that strongly mediate behavioural expression (Krause & Ruxton, 2002). Across aquatic and terrestrial systems, larger groups can often reduce individual predation risk through dilution and the ‘safety in numbers’ effect, enhance predator detection via collective vigilance, and improve foraging efficiency through social facilitation and information sharing. These changes in behavioural expression have been demonstrated in flocking birds, ungulate herds and social insect colonies (Creel & Winnie Jr, 2005; Krause et al., 2015). Thus, group size is a key determinant of behavioural expression and performance across many social taxa. In marine ecosystems, shoaling often confers comparable advantages: reef fishes in larger shoals experience reduced predation risk, greater foraging efficiency, and faster decision‐making (Pacher et al., 2025; Pitcher, 1993; Tiddy et al., 2024; Ward & Webster, 2016). Hence, whether group size, an often‐overlooked contributor to individual performance, can mediate or limit the direct effects of climate stressors on social animals has seldom been addressed.

Climate change threatens to erode group living benefits by reshaping the conditions under which shoals form and persist (Mitchell et al., 2022; Tiddy et al., 2024). Habitat degradation and ecological filtering under ocean acidification can alter fish densities (Priest et al., 2024), because the loss of structurally complex coral and macroalgal habitats to turf‐dominated assemblages reduces shelter availability (Agostini et al., 2021; Priest et al., 2024) and alters prey communities (Nagelkerken et al., 2016). These changes can suppress recruitment and survival (Nagelkerken et al., 2016), leading to local population declines that, in turn, may limit opportunities for individuals to aggregate into large shoals. Warming and hypoxia can also disrupt cohesion and coordination within shoals by altering metabolic and locomotor performance (Killen et al., 2017; Mitchell et al., 2022), while marine heatwaves could impose acute energetic demands that can shift priorities towards immediate energy acquisition at the expense of social cohesion. If shoals become smaller or less cohesive under climate change, individuals may lose the collective advantages of group living, leaving them more vulnerable to predation and less efficient in resource acquisition. Yet, despite the central role of shoaling in reef fish ecology and the generality of group size effects across animal taxa, we lack empirical tests of how climate stressors and social context interact to shape behavioural expression in the wild.

Reefs along natural temperature and CO_2_ gradients and intermittently exposed to moderate or strong marine heatwaves provide a powerful natural test of how social context governs behavioural resilience to climate extremes. We propose that climate change does not simply alter fish behaviour through physiological stress but reshapes the ecological and social environments that underpin behavioural expression. Theory predicts that climate stressors can act (i) directly, by elevating metabolic demands and shifting risk–reward trade‐offs under ocean warming and during heatwaves (Brown et al., 2004), and (ii) indirectly, by simplifying benthic habitats under acidification (Agostini et al., 2021; Priest et al., 2024), which can alter population densities (Nagelkerken et al., 2016; Priest et al., 2024) and may therefore limit the formation of larger shoals. Theory and empirical evidence on collective behaviour in animals likewise predict strong performance advantages of larger groups: by diluting predation risk and enhancing collective vigilance, accelerating information transfer and decision making, and by improving foraging efficiency, bigger groups sustain higher activity and feeding and lower refuge use (Killen et al., 2017; Krause & Ruxton, 2002; Pitcher, 1993; Ward & Webster, 2016). We hypothesised that shoal size mediates behavioural expression between foraging and predator vigilance behaviours under climate change. Specifically, we predicted that individuals in larger shoals would allocate more time to foraging and less to risk‐avoidance behaviours, consistent with theoretical expectations of predator dilution and collective vigilance (Krause & Ruxton, 2002; Pitcher, 1993). Conversely, at acidified reefs characterised by simplified habitat structure and reduced fish densities (Agostini et al., 2021), smaller shoal sizes were expected to shift behavioural allocation towards increased predator vigilance and reduced foraging. Under this framework, we tested whether climate‐driven habitat restructuring alters behavioural expression indirectly by modifying shoal size rather than through direct effects of warming, acidification or marine heatwaves.

## 2 MATERIALS AND METHODS

### 2.1 Methodological overview

To test whether shoal size mediates behavioural expression under ocean warming, acidification, and marine heatwaves, we combined field‐based behavioural observations, demographic surveys, habitat assessments and prey availability measurements across natural climate analogue reefs in Japan. These reefs represent present‐day (Control), ocean warming (OW) and combined ocean warming and acidification (OAW) conditions.

Between 2021 and 2024, we quantified focal individual behaviours of *Pomacentrus coelestis* during non‐heatwave, moderate and strong marine heatwave periods using in situ video recordings. We measured foraging, activity and risk‐avoidance behaviours and related these responses to shoal size and reef condition.

We also conducted standardised visual transect surveys to quantify focal fish densities, measured benthic habitat structure to assess reef complexity, and quantified zooplankton abundance as a proxy of food availability. Seawater temperature and carbonate chemistry were characterised concurrently to define environmental conditions.

Our experimental design allowed us to evaluate both direct climatic effects on behaviour and indirect effects mediated through habitat simplification, prey availability and changes in shoal size and population density.

### 2.2 Study species

To assess how behavioural expression is altered by acute (marine heatwaves) and chronic climate stressors (ocean warming and acidification), we selected the neon damselfish (*Pomacentrus coelestis*) as our focal species, defined here as the species selected for targeted behavioural observation and analysis. *P. coelestis* naturally forms shoals and is a habitat generalist (Beck et al., 2017; Matis et al., 2018), making it an ideal model for testing how social context (shoal size) modulates behavioural expression under both chronic and acute climatic conditions. *P. coelestis* is an omnivore, feeding predominantly on plankton and some benthic algae (Kingsford et al., 2017).

The focal species' site‐attached nature and high local densities enabled robust quantification of key behavioural metrics, including foraging, swimming activity, time near refugia and risk‐related responses across contrasting climatic conditions.

### 2.3 Study sites

**FIGURE 1:**
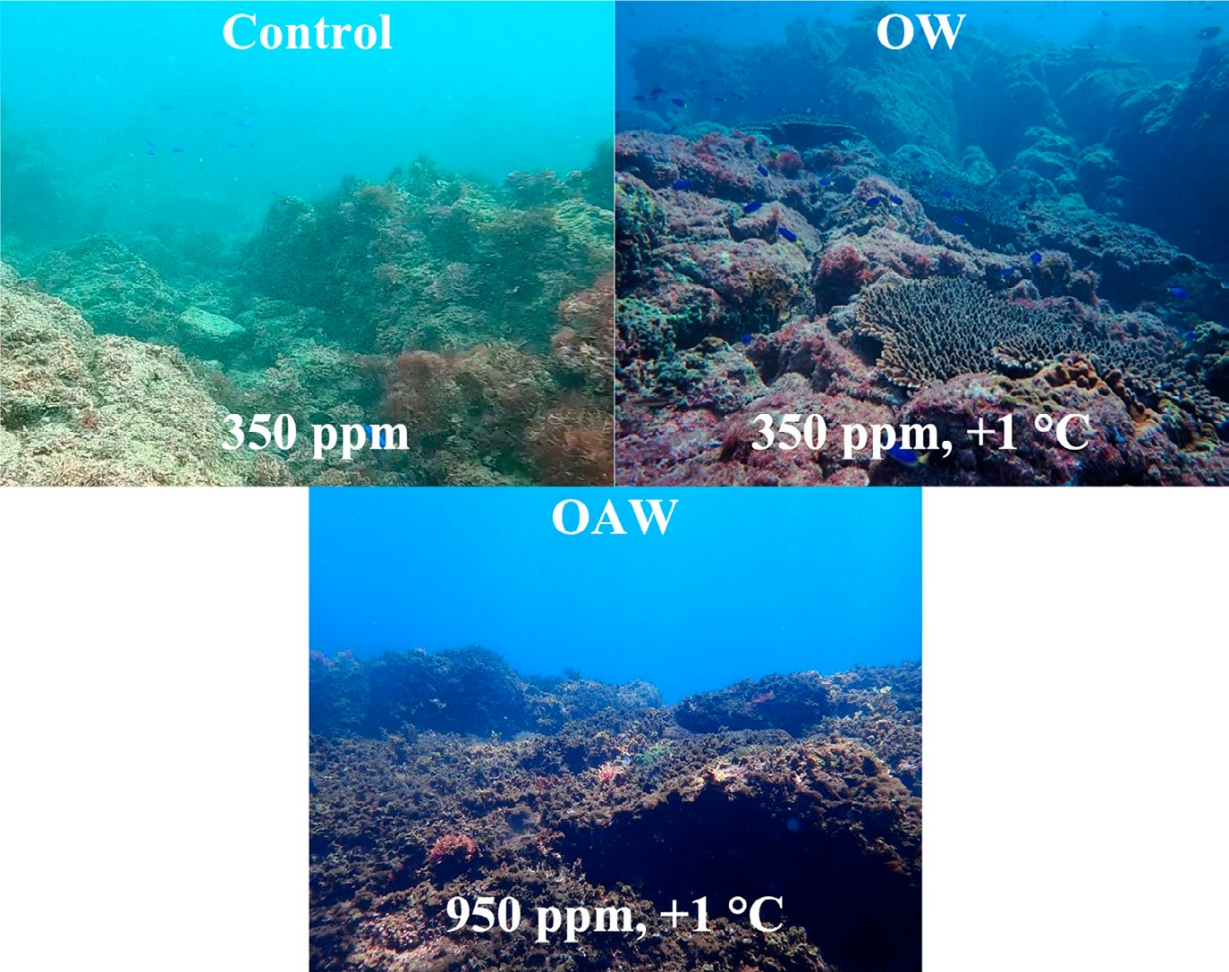
Underwater images of the three reefs used in this study, representing a natural gradient of climate change exposure. The control reef (top left) is characterised by a mosaic of canopy‐forming macroalgae, erect calcareous algae and crustose coralline algae. The ocean warming (OW) reef (top right), influenced by the Kuroshio Current, experiences elevated annual temperatures (~ +1°C) and supports a benthic community comprised of hard and soft corals, turf algae and some fleshy macroalgae. The combined ocean warming and acidification (OAW) reef (bottom) is highly acidified (~950 ppm CO_2_), dominated by a benthic community covered by a turf algae complex.

We conducted observations and densities at three nearshore reefs located in Shizuoka and Tokyo Prefectures, Japan, at the leading‐range edge of *P. coelestis*. One reef represented a current‐day warm‐temperate reef ecosystem (“Control”) situated off the Izu Peninsula (34.665051, 138.945387). Two additional reefs located near Shikine Island (~50 km from the control reef) served as natural climate analogues (Hayes et al., 2026). The ocean warming reef (hereafter: ‘OW reef’; 34.318142, 139.211097) experiences ~1°C higher annual mean sea temperature than the Control reef, driven by the influence of the Kuroshio Current (Murazaki et al., 2015). The ocean warming and acidification reef (hereafter: ‘OAW reef’; 34.321872, 139.203848) experiences acidification consistent with SSP5 projections due to a localised volcanic CO_2_ seep (Table S1). Unlike some volcanic vent systems, the CO_2_ seep sites at Shikine Island are not contaminated by heavy metals or other confounding chemical pollutants, making them suitable natural analogues for studying ocean acidification in isolation (Agostini et al., 2018; Harvey et al., 2019). The control reef is characterised by a benthic community of canopy‐forming macroalgae, erect calcareous algae and crustose coralline algae (CCA) covering rocky substratum (Figure 1; Agostini et al., 2021). In contrast, the OW reef is dominated by turf algae, some fleshy macroalgae and an increasing presence of (sub)tropical coral species which cover ~10%–15% of the OW reef's benthos (Figure 1; Cattano et al., 2020). The OAW acidification reef is structurally simplified and dominated by homogeneous diatom turf algae (>90% benthic cover), with limited coral (<1% total cover) and macroalgal cover (<5% of total benthic cover; Figure 1; Agostini et al., 2018, 2021; Harvey et al., 2019).

### 2.4 Seawater chemistry

Seawater chemistry at the Control, ocean‐warming (OW) and ocean warming and acidification (OAW) reefs has been characterised across multiple years and seasons in previous studies (Agostini et al., 2015, 2018, 2021; Cattano et al., 2020; Harvey et al., 2019). During the present study, salinity, temperature and pH were measured in situ on days when behavioural video observations were conducted in November 2022, June–July 2023 and May 2024 (*n* = 3–5 measurements per reef per sampling period).

Total alkalinity values reported by Agostini et al. (2021) and Cattano et al. (2020) were used to calculate seawater *p*CO_2_ for each reef locality using CO2SYS for Excel (Pierrot et al., 2006), with carbonate system dissociation constants from Mehrbach et al. (1973) refitted by Dickson and Millero (1987). Seawater chemistry data for all three study reefs are summarised in Table S1.

### 2.5 Marine heatwave characteristics across reef localities

Following Hobday et al. (2016), we define marine heatwaves as events lasting at least five consecutive days during which sea temperatures exceed the 90th percentile relative to a 30‐year climatological baseline, categorised as Category I ‘moderate’ (+1–2°C) or Category II ‘strong’ (+2–3°C) following Hobday et al. (2018).

Between 2021 and 2024, three Category I (moderate) and one Category II (strong) marine heatwave events were experienced across the study reefs (Figure S1; Table S2). No heatwave was detected during the 2021, June 2023, or May 2024 survey periods. Behavioural observations in May and August 2022 coincided with moderate heatwave events, and November 2022 observations coincided with a third moderate heatwave (25 October–20 December 2022; peak 4 November; mean +2.28°C; max +2.67°C; cumulative 130.11°C·days). In July 2023, observations at the OW and OAW reefs coincided with a Category II strong heatwave (max +3.51°C; cumulative 78.87°C·days). At the Control reef, surveys on 14 July 2023 occurred 9 days prior to the formal strong heatwave onset (23 July 2023), but under anomalously warm conditions (+2.5°C above climatological mean); these were therefore treated as strong heatwave conditions in the analyses. Full heatwave characteristics are summarised in Table S2.

### 2.6 Fish behaviour data collection

To quantify fish behaviour during non‐heatwave, moderate and strong marine heatwave periods, in situ video observations were conducted across natural climate‐change analogue reefs between July 2021 and May 2024. Behavioural observations were carried out in July 2021; May, August and November 2022; June and July 2023; and May 2024 (Figure S1). Researchers on SCUBA deployed 4–8 GoPro Hero cameras on the benthos at depths of 2–8 m across Control, ocean warming (OW), and ocean warming and acidification (OAW) reefs per dive. Cameras were positioned at least 10 m apart to ensure independent observations and were placed approximately 1–2 m from naturally occurring shoals of *P. coelestis* encountered across each reef. Camera placements were distributed across reef areas to avoid repeatedly sampling the same shoals. To minimise diver disturbance, the first and last 2 min of each recording were excluded from analyses. Usable focal‐fish observation periods ranged from 30 s to 15 min (mean observation time ~4 min). Mean (±SD) observation durations were 315 ± 317 s at the Control reef, 193 ± 174 s at the OW reef, and 261 ± 192 s at the OAW reef.

### 2.7 Fish behaviour analysis

All behaviours of a focal *P. coelestis* individual were analysed for each recording using Windows Media Player on a desktop computer. Video recordings were a maximum of 15 min in duration. Because individuals occasionally moved in and out of the camera's field of view, focal observations were terminated when a fish exited the frame and were not resumed if the individual re‐entered.

For each video, behaviours were recorded from a single focal individual. When only one individual was present within the field of view, that individual was designated as the focal fish. When multiple individuals were present, the individual closest to the centre of the screen was designated as the focal fish. If the selected individual did not remain within the field of view for at least 30 s, the next closest individual to the selected grid cell was chosen.

Behavioural observations were quantified from video recordings by undergraduate research assistants who were trained in the use of standardised ethograms and scoring protocols prior to data collection. Observers were not involved in study design, fieldwork, hypothesis development or data analysis and were unaware of predicted behavioural responses across reefs or marine heatwave conditions. Scored datasets were subsequently validated by senior authors through cross‐checking a subset of videos (five per reef) and associated data entries to confirm scoring consistency and data accuracy.

The following four behaviours were quantified for all focal species in the video recordings: (1) bite rate, continuously counted during the observation and measured as the total number of bites taken by the focal species on the benthos and in the water column at potential prey items (e.g. zooplankton); (2) retreat rate, continuously counted and measured as the number of times a fish rapidly retreated away from the water column towards shelter or a shoal of conspecific fishes, before returning to its previous position; (3) activity levels, quantified as the percentage of total observation time during which a focal fish was actively moving in the water column; (4) time near refugia, quantified as the percentage of total observation time a fish resided within five body lengths of substrate shelter. All behaviours that were measured as continuous counts (bite and retreat rates) were expressed as rates per unit of time (e.g. behaviours·min^−1^). A total of 91 fish‐behavioural recordings were analysed across reefs and heatwave conditions. Replication for behavioural responses across reef and heatwave conditions are reported in Table 1.

**TABLE 1:** Sample sizes (*n*) for behavioural observations, flight initiation distance (FID) measurements and population density surveys for each combination of reef location and marine heatwave status.

Behavioural proxies	Marine heatwave status		
Reef	Non‐heatwave	Moderate	Strong
Control	0	10	2
OW	30	23	2
OAW	9	12	3

Flight initiation distance	
Reef	Strong
Control	15
OW	19
OAW	5

Population density surveys		
Reef	Non‐heatwave	Strong
Control	10	10^a^
OW	10	10
OAW	10	10

*Note*: Behavioural observations were conducted in July 2021; May, August, and November 2022; June and July 2023; and May 2024. Flight initiation distance responses were measured only in July 2023, during the strong marine heatwave that occurred at all studied reefs. Population density surveys were conducted during the strong marine heatwave in July 2023 and during non‐heatwave conditions in May 2024.

^a^ Density surveys at the Control reef occurred approximately 9 days before the commencement of the strong heatwave event and should be considered as an acute warming anomaly (~ +2.5°C above climatological mean) rather than a strong heatwave event.

### 2.8 Flight initiation distance

During the strong marine heatwave event, between 25 and 30th July 2023, we quantified the predator escape behaviour of *P. coelestis* using a standardised flight initiation distance (FID) test across three reef types (Control, OW, OAW) to assess how predator threats were perceived under future climate scenarios and across varying shoal sizes. We defined FID ‘as the distance at which a focal fish initiated an escape response from the approaching artificial threat’. An artificial threat stimulus was constructed from a cubical PVC frame attached to a 60‐cm iron rod that supported a 30‐cm metal ruler at its distal end, following Mitchell et al. (2025). A GoPro Hero 7 Silver camera was mounted on the PVC frame and directed towards the ruler to capture behavioural responses. Once a *P. coelestis* was located, a diver slowly advanced the ruler tip towards the fish's head at a constant speed while recording its response (frame rate: 30 fps; Figure S2). We also recorded the shoal size (*n*) of which the focal fish was a member of.

To minimise non‐independence of replicates, only one individual was sampled per shoal, and observations were restricted to a single 60–90 min dive per reef. FID was calibrated against the attached ruler and quantified to the nearest 0.5 cm. We measured FID in 15 fish at the control reef, 19 at the OW reef, and 5 at the OAW reef, with one individual sampled per shoal to minimise non‐independence.

### 2.9 Fish density surveys

Fish density surveys were conducted using SCUBA‐based belt transects. A diver swam along a 10 m transect, recording the densities of *P. coelestis* individuals within 2 m on each side of the transect tape (survey area = 40 m^2^ per transect) at depths of 5–8 m.

In addition to density surveys, total length (cm) of each individual was visually estimated in situ to the nearest centimetre using the transect tape as a direct size reference, following standard underwater visual census (UVC) protocols widely used in reef fish ecology (Edgar et al., 2004). The transect line provided a fixed, scaled reference in the diver's field of view to improve estimation consistency. The focal species body length ranged between 3 and 8 cm total length, and centimetre‐scale resolution provides sufficient precision for detecting reef‐level differences in mean body size within this size range (Colvocoresses & Acosta, 2007; Wilson et al., 2018).

Surveys were conducted at the Control reef on 14 July 2023 under non‐heatwave conditions (*n* = 10 transects), and at the ocean‐warming (OW) and ocean warming and acidification (OAW) reefs during a strong marine heatwave (25–28 July 2023; *n* = 10 transects per reef) and again under non‐heatwave conditions at all reefs (19–24 May 2024; *n* = 10 transects per reef). Density data from each transect were converted to mean density (individuals per 40 m^2^) for each reef and heatwave period.

Fish density surveys were not conducted during the 2022 moderate marine heatwave period.

### 2.10 Habitat complexity

Because the focal species *P. coelestis*' broad habitat use depends on what is locally available (Soeparno et al., 2013; Matis et al., 2018), we quantified habitat complexity across the Control, Ocean Warming (OW) and Ocean Warming + Acidification (OAW) reefs by measuring the mean maximum canopy height of benthic vegetation. At each reef in May 2024, the maximum height (cm) of the dominant canopy‐forming or turf algae was measured using a ruler positioned vertically from the substrate to the canopy tip of a randomly selected point. A total of 50 measurements were taken at the Control reef, 45 at the OW reef, and 35 at the OAW reef. The mean maximum canopy height per reef was then used as a proxy for benthic structural complexity.

### 2.11 Food availability

To test whether food availability influenced shoal size and behavioural expression in *P. coelestis*, we quantified zooplankton abundance—its predominant dietary item (Kingsford et al., 2017)—at each reef in July 2023. Zooplankton were sampled using a plankton tow net (mouth area ~707 cm^2^), which was deployed vertically from the research vessel to 5 m depth above each reef (*n* = 10 tows per reef). After retrieval, samples were filtered through Whatman GF/C glass fibre filters (0.7 μm pore size) and preserved in 70% ethanol.

In the laboratory, zooplankton were rinsed from the filters with deionised water and examined under a stereomicroscope. All individuals in each sample were counted. To facilitate comparison across reefs, zooplankton densities were standardised to units per m^2^ based on the net mouth area and the vertical tow distance. Reef‐level mean zooplankton densities were calculated from the 10 independent net hauls per reef. Percentage differences among reefs reported in the Results refer to percentage (%) increase or decrease between reef‐level mean densities.

### 2.12 Statistical analysis

All statistical analyses were conducted in R version 4.4.0 (R Core Team, 2024). Generalised linear mixed‐effects models (GLMMs) and generalised linear models (GLMs) were used to test the effects of reef type and marine heatwave (MHW) category on shoaling behaviour, shoal size, and population density of *Pomacentrus coelestis*. Models were fitted using the *glmmTMB* package (Brooks et al., 2017), which allows flexible specification of non‐Gaussian error distributions and random effects.

Behavioural responses (bite rate, retreat rate, activity levels, and time spent near refugia) and shoal size were analysed using GLMMs. Year was included as a random intercept to account for repeated sampling and temporal non‐independence. Fixed effects included reef type (Control, OW, OAW), MHW category (non‐heatwave, moderate, strong) and shoal size (where appropriate).

Error distributions were selected based on the statistical properties of each response variable. Bite rate, retreat rate and time spent near refugia exhibited skewness and zero inflation and were therefore modelled using a Tweedie distribution with a log link. Activity levels, expressed as percentages, were modelled using a Gaussian distribution with a log link, which stabilised variance and ensured positive fitted values. Shoal size was analysed using a negative binomial distribution with a log link to account for overdispersion relative to a Poisson model.

Model adequacy was assessed using simulation‐based residual diagnostics implemented in the *DHARMa* package (Hartig, 2022), including tests for uniformity, overdispersion, and zero inflation. When model assumptions were violated, the error distribution, link function, or fixed effect structure was adjusted to improve model fit, and the most parsimonious model with the best residual diagnostics was retained as the final model for each response variable. Significance of fixed effects was assessed using Type III Wald χ^2^ tests implemented in the car package (Fox & Weisberg, 2019). For factors with more than two levels (reef type and MHW category), post hoc pairwise comparisons of estimated marginal means were conducted using Tukey‐adjusted contrasts in the *emmeans* package (Lenth, 2024).

To evaluate the effects of reef type, MHW category and their interaction on the density of *P. coelestis*, a GLM with a Tweedie error distribution and log link was fitted. Model significance was assessed using Type III Wald *χ* ^2^ tests, and significant terms were explored using Tukey‐adjusted post hoc comparisons of estimated marginal means.

To assess differences in body size across reefs and heatwave periods, individual total length (cm) of *P. coelestis* was analysed using transect survey data. Transect‐level counts were expanded so that each fish was treated as an individual observation, while transect identity was included as a random intercept to account for non‐independence of fish within transects. Total length was modelled using a GLMM with reef type (Control, OW, OAW), MHW category (non‐heatwave, strong) and their interaction as fixed effects, assuming a Gaussian error distribution with a log link. Model assumptions were evaluated using DHARMa residual diagnostics, and significance of main effects and interactions was assessed using Type III Wald *χ* ^2^ tests with Tukey‐adjusted post hoc comparisons.

Differences in zooplankton abundance and mean maximum canopy height among reefs were analysed using Kruskal–Wallis tests, with reef type treated as a fixed factor. This non‐parametric approach was used because assumptions of normality and homogeneity of variance were violated, as assessed using Shapiro–Wilk and Levene's tests. When significant effects were detected (*p* < 0.05), pairwise Wilcoxon rank‐sum tests with Holm correction were used to identify differences among reef types.

## 3 RESULTS

### 3.1 Shoal size increases behavioural expression across climate scenarios and heatwaves

**FIGURE 2:**
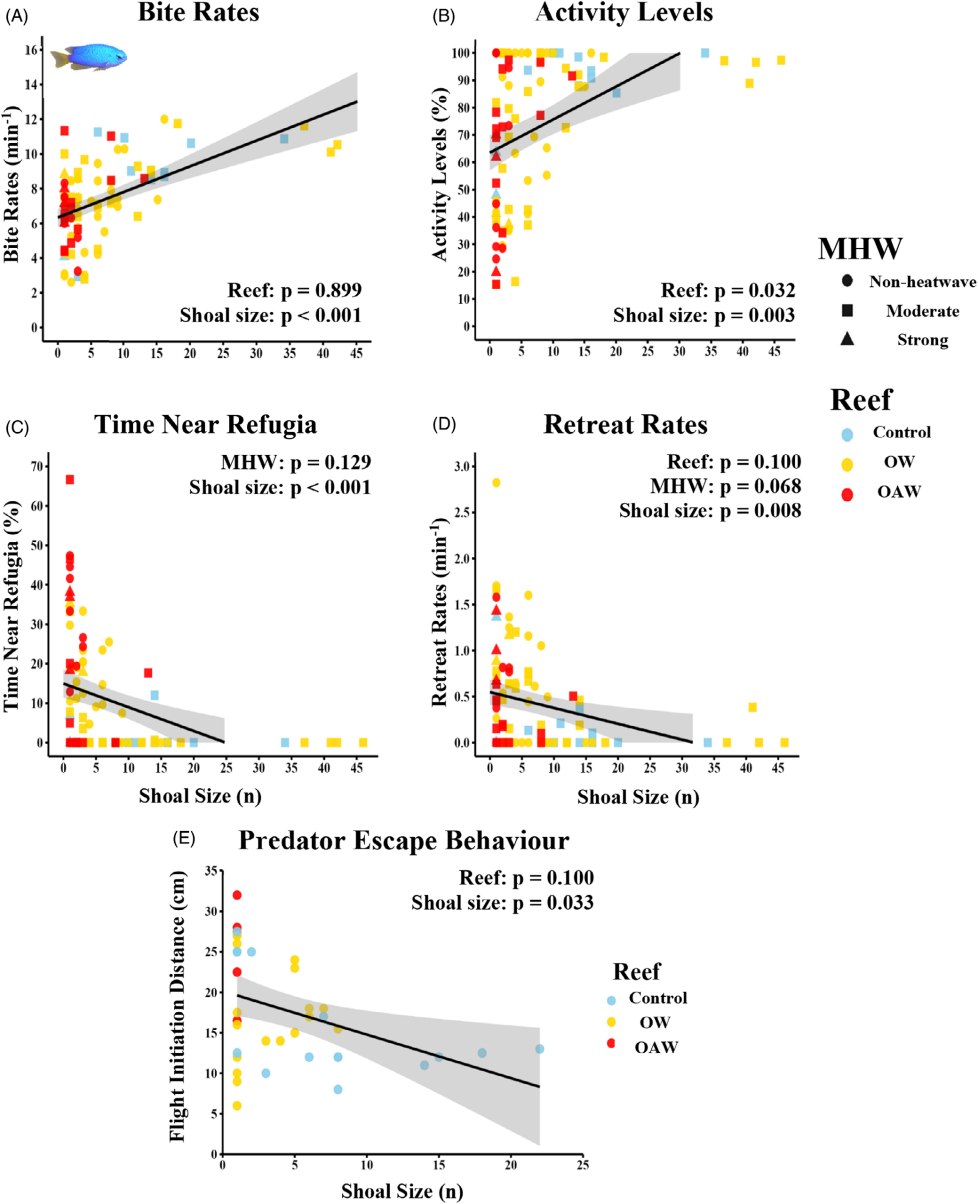
Behavioural responses of focal reef fish across shoal sizes, reef locations, and marine heatwave (MHW) conditions. Relationships between shoal size and (A) bite rates, (B) activity levels, (C) time near refugia, (D) retreat rates and (E) flight initiation distance (FID) across reefs (Control = blue, Ocean warming = yellow, Ocean acidification + warming = red) and marine heatwave conditions (Non‐heatwave = circles, Moderate = squares, Strong = triangles). Lines show fitted model predictions with 95% confidence intervals (grey shading). FID was measured only during the strong marine heatwave (July 2023) and is therefore shown separately in panel (E). Statistical outputs for final behavioural models are provided in Tables S3–S7.

Bite rates and activity levels of fish increased with increasing shoal size (Figure 2a,b; bite rates: estimate = 0.015 ± 0.003 SE; *χ* ^2^ = 27.80, *p* < 0.001; activity levels: estimate = 0.008 ± 0.003 SE; *χ* ^2^ = 8.61, *p* = 0.003), while retreat rates (Figure 2c,d; estimate = −0.068 ± 0.025 SE; *χ* ^2^ = 7.11, *p* = 0.008), flight initiation distance (Figure 2e; estimate = −0.027 ± 0.013 SE; *χ* ^2^ = 4.57, *p* = 0.033), and time spent near refugia (estimate = −0.210 ± 0.048 SE; *χ* ^2^ = 19.33, p < 0.001) decreased as shoal size increased, irrespective of ocean warming, acidification and marine heatwave type across all reefs (Tables S3–S7).

Bite rates, retreat rates, time near refugia and flight initiation distance were unaffected by marine heatwaves across reefs experiencing ocean warming and/or acidification (*χ* ^2^ = 1.54, *p* = 0.463; *χ* ^2^ = 0.21, *p* ≤ 0.899; *χ* ^2^ = 4.61, *p* = 0.100, respectively; Tables S3, S4, S6, S7). However, fish activity levels were 22% lower at the combined stressor (OAW) reef than at the Control reef (estimate = −0.25 ± 0.10 SE; *p* = 0.012; Table S5).

### 3.2 Ocean acidification and marine heatwaves alter shoal size and population structure, but not fish body size

Fish body size did not differ across reefs (Control–OW: *p* = 0.352; Control–OAW: *p* = 0.998; OW–OAW: *p* = 0.058) or heatwave type (*p* = 0.576, Figure S3; Table S10).

**FIGURE 3:**
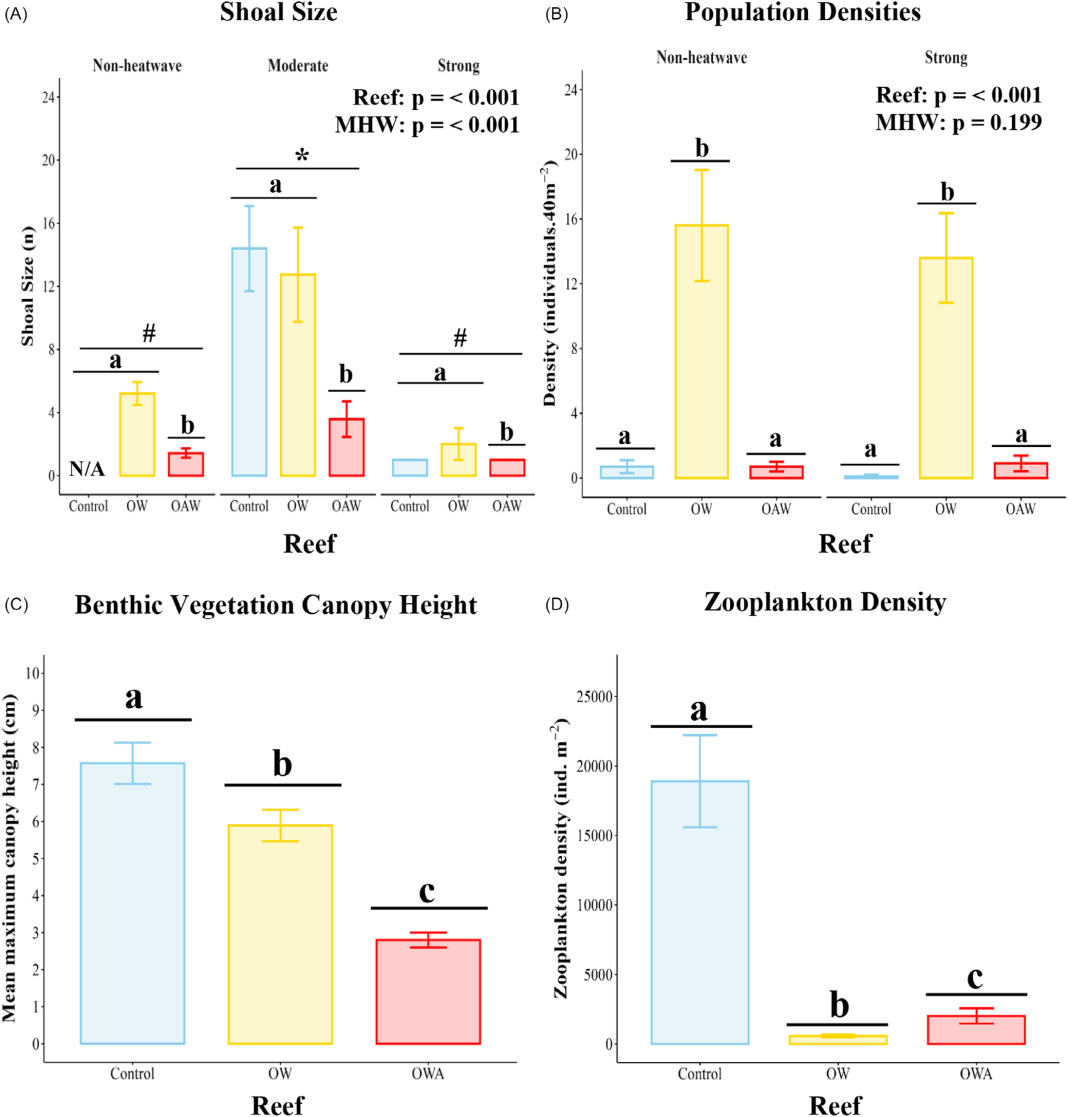
Shoal size, population density, and associated habitat resources of *Pomacentrus coelestis* across reefs and marine heatwave periods. (A) Mean shoal size (±SE) across reefs representing Control (blue), ocean warming (OW; yellow), and combined ocean warming and acidification (OAW; red) conditions during non‐heatwave, moderate, and strong marine heatwave (MHW) periods. Shoal sizes differed significantly among reefs (*p* < 0.001) and among MHW type (*p* < 0.001). N/A denotes no video‐based shoal size sampling during non‐heatwave periods at the Control reef. (B) Mean population density (±SE; individuals per 40 m^−2^) across reefs and MHW type. Different letters denote significant pairwise differences among reefs (Tukey post hoc tests, *p* < 0.05), while symbols (#, *) indicate significant differences between MHW type across reefs. (C) Mean (±SE) maximum canopy height of benthic vegetation across reefs. (D) Mean (±SE) zooplankton density (individuals m^−2^) across reefs. For panels (C) and (D), different letters indicate significant differences among reefs based on Kruskal–Wallis tests followed by pairwise Wilcoxon rank‐sum tests with Holm correction (*p* < 0.05). Full statistical outputs are provided in Tables S8, S9, S11 and S12.

Shoal sizes were similar at the Control and OW reefs, but 70%–79% smaller at the OAW reef (Figure 3A, contrast estimate for control: −1.27 ± 0.32 SE; *p* < 0.001; for OW: −1.21 ± 0.24 SE; *p* < 0.001). Shoal sizes were 145% larger during moderate heatwaves than during non‐heatwave periods (contrast estimate = 0.90 ± 0.20 SE; *p* < 0.001) and 730% larger than during strong heatwaves, irrespective of reef type (1.88 ± 0.47 SE; *p* < 0.001; Figure 3B; Table S8).

In contrast, fish population densities were similar at the Control and OAW reefs (*p* = 0.422; Figure 3B; Table S9) but were 84% lower than at the OW reef (contrast estimate for OW–OAW = 2.91 ± 0.46 SE; Control–OW = −4.01 ± 0.77 SE; *p* < 0.001). Population densities (*χ* ^2^ = 1.65, *p* = 0.199) were unaffected by the strong heatwave event (Table S9).

### 3.3 Indirect effect of ocean acidification on habitat complexity

At the OAW reef, mean maximum canopy height of benthic vegetation was 63% lower than the Control reef (Figure 3C; *p* < 0.01; Table S11) and 52% lower than the OW reef (*p* < 0.01). Additionally, canopy height was 22% lower at the OW reef than the Control reef (*p* = 0.022).

### 3.4 Food availability across climate scenarios

Zooplankton densities were 3174% higher at the Control reef than the OW reef (Figure 3D; *p* < 0.001) and 839% higher at the Control reef than the OAW reef (*p* < 0.001). The OAW reef also had 249% higher zooplankton densities than the OW reef (*p* = 0.025; Table S12).

## 4 DISCUSSION

Our findings support the hypothesis that shoal size mediates behavioural expression in a range‐extending fish (*Pomacentrus coelestis*). Across reefs representing control, ocean warming, and combined warming and acidification conditions, shoal size consistently predicted behavioural expression, whereas direct exposure to marine heatwaves, ocean warming, and acidification had limited effects on individual behaviour. Individuals in larger shoals allocated more time to foraging and activity and less to retreat and refuge use, irrespective of climate conditions or food availability. These patterns are consistent with theoretical expectations that group living modifies risk allocation through predator dilution, collective vigilance, and social facilitation (Foster & Treherne, 1981; Krause et al., 2015; Krause & Ruxton, 2002). While group living is often associated with ecological and fitness advantages across taxa (Creel & Winnie Jr, 2005), our results do not directly quantify survival or physiological performance. Rather, they demonstrate that shoal size mediates behavioural expression under ocean warming, acidification and marine heatwaves. Combined, our findings suggest that collective behaviour is an important mediator of behavioural expression in reef fishes and may influence how individuals respond to climate‐driven environmental change.

We found that the indirect effects of ocean acidification seem stronger than the direct effects as they constrain collective behaviour by simplifying benthic habitats and reducing opportunities for large‐shoal formation. At the combined warming and acidified (OAW) reef, canopy‐forming vegetation was more than 50% shorter than at the OW and control reefs, whilst hard corals were nearly entirely absent (Cattano et al., 2020), reflecting strong habitat simplification and a loss of structural refugia commonly observed on acidified reefs (Agostini et al., 2021; Priest et al., 2024). Reduced habitat complexity and near absence of hard corals (reported in Cattano et al., 2020) coincided with 84% lower focal fish densities than at the OW reef and 70%–79% smaller shoals than at the control and OW reefs. In contrast, differences in food availability across reefs did not align with lower densities and shoal sizes; the OAW reef had 249% higher zooplankton densities than the OW reef. Food availability therefore cannot explain the observed reductions in shoal size under acidification, reinforcing a loss of habitat complexity as the important driver of shoal size reductions. Lower predator densities reported at the acidified reef (Cattano et al., 2020), together with higher prey availability, may also have influenced shoal formation. Reduced predation pressure could lessen the relative advantage of maintaining large aggregations, while elevated local food availability may reduce the need for collective foraging and intra‐shoal competition in areas where resources are readily accessible (Reuter et al., 2016). These mechanisms may contribute to shoal‐size variation, although they do not readily account for the pronounced reduction in focal fish density coincident with habitat simplification. Therefore, we suggest the loss of canopy height and coral complexity reduces refuge availability and may elevate either direct or perceived predation risk, because smaller shoals must invest more in vigilance, collective surveillance becomes costlier, shifting time budgets away from foraging and reducing activity (Pitcher, 1993). Fish within smaller shoals on the acidified reef were indeed less active than at the control and OW reefs. Smaller shoals are inherently less cohesive and coordinated than larger shoals, so individuals spend more time on vigilance and predator detection (Pitcher, 1993; Ward & Webster, 2016). Diminished collective vigilance likely explains the lower activity observed in *P. coelestis* under acidification. Our findings reveal an indirect, socially‐mediated pathway of climate impacts driven by habitat simplification under ocean acidification. We therefore conclude that ocean acidification can indirectly alter behavioural expression in shoaling fish by eroding the ecological and social conditions that support collective behaviour.

The direct effects of various climate stressors on fish behaviours appear to be minimal for range extending fish. The focal shoaling fish had largely similar behaviour across natural analogues of ocean warming and acidification (except for activity levels), as well as no detectable changes in foraging, activity, or risk‐avoidance behaviours in response to marine heatwaves. The near absence of direct behavioural effects underlines the importance of ecological context in shaping species responses to climate extremes. We note that our study relies on a comparison of natural climate analogues rather than a fully factorial experimental control framework. All reefs experience contemporary thermal regimes characteristic of the species' leading range margin; therefore, our inferences are relative among reefs and heatwave intensities rather than against a historical thermal baseline. Behavioural stability observed here should thus be interpreted as context‐dependent within the present‐day thermal envelope of these populations. The 2023 global marine heatwave, although one of the most intense on record (Dong et al., 2025), likely did not exceed the thermal optimum of *P. coelestis* at our study location. Our study was conducted near the cooler, temperate margin of the species' distribution in Japan, where background temperatures are lower than in its tropical core range. Under these conditions, even the observed marine heatwave events remained within the species' preferred thermal range, allowing individuals to maintain normal behavioural expression relative to non‐heatwave conditions. However, shoal size varied across heatwave intensities. Shoals were larger during moderate heatwave conditions but declined again during strong heatwave periods. This pattern suggests that moderate thermal anomalies at the species' cooler range margin may temporarily enhance aggregation, potentially increasing the social context within which behaviours are expressed, whereas stronger heatwaves do not sustain this effect. Thus, while marine heatwaves did not directly modify behavioural expression at the individual level, they may influence behavioural dynamics indirectly by altering group size and population density. While our study cannot directly assess range‐extension related mechanisms, our findings suggest that biogeographic context is important to understanding behavioural responses to climate change, particularly at species' leading range edges where temperatures may be a limiting factor of performance (Coni et al., 2022; Hayes et al., 2024, 2025; Mitchell et al., 2025). The magnitude and direction of behavioural adjustments are likely to differ among populations according to local thermal regimes and ecological settings, meaning that the same climatic event may elicit contrasting outcomes across a species' range (Donelson et al., 2019). For example, a +3°C temperature increase within the core tropical ranges of *P. coelestis* has been shown to reduce metabolic and swimming performance (Johansen & Jones, 2011), whereas the strong marine heatwave (~ + 3.5°C) we observed at the focal species cooler range margin showed negligible effects on behavioural expression metrics. Consequently, we posit that the role of social context in shaping fish behaviour can vary geographically, reflecting how local climatic conditions and population structure interact to influence the expression of behaviours in shoaling species at their range edges.

By integrating natural analogue systems with field observations of collective behaviour, our study identifies a socially mediated pathway governing behavioural expression in shoaling fishes under acute and chronic climate stressors. Shoal size was the proximate determinant of foraging, activity, and risk‐avoidance behaviours across all reefs, with larger shoals consistently experiencing enhanced behavioural expression. Ocean acidification did not act by directly altering behaviour, but by constraining this social mechanism indirectly. At the acidified reef, acidification‐driven habitat simplification likely reduced fish densities and thereby limited the formation of large shoals, indirectly altering behavioural expression. Hence, our data show that ocean acidification does not directly modify behaviour in our study species but indirectly alters behavioural expression by altering habitat complexity and social structure in ways that limit the formation of large shoals. Behavioural expression is therefore not solely an intrinsic individual trait, but an emergent property of the social–ecological context in which individuals are embedded. Therefore, we reframe shoaling not simply as a buffer against environmental stress, but as a socially mediated process whose behavioural expression depends on habitat structure and population density under ocean warming and acidification.

## 5 CONCLUSIONS

We conclude that increasing shoal size can mediate behavioural expression in shoaling fish exposed to ocean warming, acidification and marine heatwaves. However, ocean acidification indirectly modifies behavioural expression by simplifying reef habitats and reducing fish population densities, thereby limiting the formation of larger shoals. Combined, our results demonstrate that the behavioural expression of shoaling fishes under future ocean conditions is shaped not only by their individual physiological and behavioural tolerances to climate change, but by the persistence of the ecological and social contexts that support collective behaviour.

## AUTHOR CONTRIBUTIONS

Angus Mitchell and Ivan Nagelkerken conceived the study. Angus Mitchell, Ivan Nagelkerken, Mary E. Hart, Sean D. Connell, Ben P. Harvey, Sylvain Agostini, Davide Spatafora, Michael Izumiyama and Timothy Ravasi conducted fieldwork and collected the data. Angus Mitchell analysed the data. Ivan Nagelkerken, Sean D. Connell, David J. Booth and Timothy Ravasi secured funding. Angus Mitchell wrote the manuscript. All authors contributed to revisions and approved the final version.

## CONFLICT OF INTEREST STATEMENT

The authors have no conflicts of interest to declare.

## ETHICS STATEMENT

All data were collected under animal ethics approvals: S‐2023‐043 (University of Adelaide) and followed universities' animal ethics guidelines. Behavioural recordings and fish surveys were conducted under Shizuoka Prefecture permit number 5–10 (2023) and Tokyo Prefecture permit number 5–11 (2023).

## Supporting information


**Figure S1.** Daily sea temperature time series at the study reef (139.125° E, 34.375° N) from January 2021 to May 2024, extracted from the Marine Heatwave Tracker (tracker.marineheatwaves.org). The solid black line shows observed daily temperatures, with shaded areas indicating periods when temperatures exceeded the long‐term seasonal baseline (marine heatwave conditions). Dashed lines represent seasonal climatologies. Vertical lines indicate periods when fish behavioural observations were conducted, coloured by marine heatwave category (black = non‐heatwave, orange = moderate heatwave, red = strong heatwave).
**Figure S2.** Example of flight initiation distance (FID) measurement for *Pomacentrus coelestis* in situ. (A) The focal fish (circled) prior to the simulated predator approach. (B) The measured distance (cm) at which the fish elicited a flight initiation response as the diver advanced the threat stimulus (metal ruler attached to a PVC frame). (C) The position of the fish immediately after initiating flight, with the red line indicating the escape direction of the fish at the point of response.
**Figure S3.** Mean (±SE) body size of *Pomacentrus coelestis* across reefs (Control, OW, OAW) and marine heatwave (MHW) periods (non‐heatwave, strong). Bars show mean total length (cm) at the individual level, with reef colours (Control = sky blue, OW = gold, OAW = red) and MHW status indicated by fill pattern (striped = non‐heatwave, solid = strong). Body size did not differ significantly among reefs in post hoc pairwise comparisons (all *p* ≥ 0.058) and was unaffected by MHW period (*χ* ^2^ = 0.31, *p* = 0.576) or the reef × MHW interaction (*χ* ^2^ = 0.28, *p* = 0.869; Appendix S1). Error bars are not shown for the Control reef during the strong heatwave, as only one individual was detected on transects during this period.
**Table S1.** Seawater chemistry across reefs in 2022, 2023, and 2024. Mean (±SD) values of seawater temperature, salinity, pH, and calculated *p*CO_2_ (μatm) at the Control, OW, and OAW reefs during November 2022 (17–18, moderate heatwave), June 2023 (8–14, non‐heatwave), July 2023 (21–27, strong heatwave), and May 2024 (19–24, non‐heatwave). Salinity and pH were not sampled at the Control reef during November 2022. Seawater chemistry data were collected concurrently with fish community surveys (*n* = 3–4 per metric per reef). Long‐term chemistry data (2011–2018) were extracted from Agostini et al. (2021). *p*CO_2_ values were calculated using in situ temperature, salinity, and pH measurements combined with total alkalinity data from Cattano et al. (2020) (OAW reef) and Agostini et al. (2021) (OW and Control reefs). Calculations were performed in CO2SYS for Excel (Pierrot et al., 2006) using constants from Mehrbach et al. (1973) refit by Dickson and Millero (1987). Long‐term temperature deltas, pH and *p*CO_2_ data long‐term pH and *p*CO₂ data (2011–2018) were extracted from Agostini et al. (2021), while for the OAW reef MHW = Marine Heatwave.
**Table S2.** Summary of survey periods, marine heatwave (MHW) status and characteristics, and data collected at each reef location (Control, OW = ocean warming, OAW = ocean warming and acidification). MHW categories follow Hobday et al. (2018): Category I = Moderate (+1–2°C above climatological mean); Category II = Strong (+2–3°C above climatological mean). MHW characteristics (mean/max intensity and cumulative intensity) are averaged across reefs. Colour shading indicates MHW status: green = no heatwave; amber = Category I (moderate); red = Category II (strong). *Control reef sampled 9 days prior to formal MHW onset; treated as strong heatwave conditions given anomalously elevated SST (+2.5°C above climatological mean) at time of sampling.
**Table S3.** Final GLMM for bite rate and resulting statistical outputs across levels of shoal size, reef location and MHW status. Models were fitted using generalised linear mixed models (GLMMs) with a Tweedie distribution (log link) in the *glmmTMB* package in R. Year was included as a random effect. Coefficients are presented as model estimates ± standard error (SE) on the log scale, alongside the Wald *z* statistic and *p*‐values. *χ* ^2^ values are from Type III Wald *χ* ^2^ tests of fixed effects. Where relevant, Tukey‐adjusted post hoc contrasts from the *emmeans* package are reported. DHARMa residual diagnostics are included to test for uniformity, dispersion, and zero inflation. Bold *p*‐values denote significant effects (*p* < 0.05). df = degrees of freedom.
**Table S4.** Final GLMM for retreat rate and resulting statistical outputs across levels of shoal size, reef location and MHW status. Models were fitted using generalised linear mixed models (GLMMs) with a Tweedie distribution (log link) in the *glmmTMB* package in R. Year was included as a random effect. Coefficients are presented as model estimates ± standard error (SE) on the log scale, alongside the Wald *z* statistic and *p*‐values. *χ* ^2^ values are from Type III Wald *χ* ^2^ tests of fixed effects. Tukey‐adjusted post hoc contrasts from the *emmeans* package are reported for MHW levels. DHARMa residual diagnostics are included to test for uniformity, dispersion, and zero inflation. Bold *p*‐values denote significant effects (*p* < 0.05). df = degrees of freedom.
**Table S5.** Final GLMM for activity levels (%) and resulting statistical outputs across levels of shoal size and reef location. Models were fitted using generalised linear mixed models (GLMMs) with a Gaussian distribution (log link) in the *glmmTMB* package in R. Year was included as a random effect. Coefficients are presented as model estimates ± SE on the log scale, with corresponding Wald z statistics and *p*‐values. *χ* ^2^ values are from Type III Wald *χ* ^2^ tests of fixed effects. Tukey‐adjusted post hoc contrasts from the *emmeans* package are reported for location. DHARMa residual diagnostics are included to test for uniformity and dispersion. Bold P‐values denote significant effects (*p* < 0.05). df = degrees of freedom.
**Table S6.** Final GLMM for refugia use (time spent near refugia, %) and resulting statistical outputs across levels of shoal size and MHW status. Models were fitted using generalised linear mixed models (GLMMs) with a Tweedie distribution (log link) in the *glmmTMB* package in R. Year was included as a random effect. Coefficients are presented as model estimates ± SE on the log scale, with Wald *z* statistics and *p*‐values. *χ* ^2^ values are from Type III Wald *χ* ^2^ tests of fixed effects. Tukey‐adjusted post hoc contrasts from the *emmeans* package are reported for MHW levels. DHARMa residual diagnostics are included to test for uniformity, dispersion, and zero inflation. Bold *p*‐values denote significant effects (*p* < 0.05). df = degrees of freedom.
**Table S7.** Final selected model (GLM, Tweedie family with log‐link) for *Pomacentrus coelestis* flight initiation distance (FID) across shoal size and reef type (Control, OW, OAW). Models presented in Table S6 are those reported in the main Results section. Bold P‐values denote significant effects (*p* < 0.05). df = degrees of freedom.
**Table S8.** Final GLMM for shoal size and resulting statistical outputs across levels of reef location and MHW status. Models were fitted using generalised linear mixed models (GLMMs) with a negative binomial distribution (log link) in the *glmmTMB* package in R. Year was included as a random effect. Coefficients are presented as model estimates ± SE on the log scale, with Wald *z* statistics and *p*‐values. *χ* ^2^ values are from Type III Wald *χ* ^2^ tests of fixed effects. Tukey‐adjusted post hoc contrasts from the *emmeans* package are reported for reef location and MHW status. DHARMa residual diagnostics are included to test for uniformity and dispersion. Bold *p*‐values denote significant effects (*p* < 0.05). df = degrees of freedom.
**Table S9.** Final GLM for *Pomacentrus coelestis* population density and resulting statistical outputs across levels of reef (Control, OW, OAW) and MHW status (Non‐heatwave, Strong). Models were fitted using generalised linear models (GLMs) with a Tweedie distribution (log link) in the *glmmTMB* package in R. Coefficients are presented as model estimates ± standard error (SE) on the log scale, with associated Wald *z* statistics and *p*‐values. *χ* ^2^ values are from Type III Wald *χ* ^2^ tests of fixed effects. Tukey‐adjusted post hoc contrasts are reported for reef location. DHARMa residual diagnostics are included to test for uniformity and dispersion. Bold *p*‐values denote significant effects (*p* < 0.05). df = degrees of freedom.
**Table S10.** Results of a generalised linear mixed‐effects model (GLMM) testing effects of reef (Control, OW, OAW) and marine heatwave (MHW) status (non‐heatwave, strong) on total length (TL, cm) of *Pomacentrus coelestis*. The model was fitted with a Gaussian error distribution and log link, with transect identity as a random intercept (*n* = 316 individuals, 21 transects). Shown are fixed effect estimates (±SE), Type III Wald *χ* ^2^ tests, Tukey‐adjusted pairwise contrasts averaged over MHW status, and DHARMa diagnostic tests for model assumptions.
**Table S11.** Results of Kruskal–Wallis and pairwise Wilcoxon rank‐sum tests comparing mean maximum canopy height (cm) among reefs.
**Table S12.** Results of Kruskal–Wallis and pairwise Wilcoxon rank‐sum tests comparing mean zooplankton density (individuals. m^−2^) among reefs.

## Data Availability

The data and code (Mitchell et al., 2026) supporting the findings of this manuscript were provided as part of the submission for review and are available in Figshare: https://doi.org/10.25909/31441369.
