# Supplementary material for: Ocean acidification, more than warming or heatwaves, constrains shoaling behaviour in a range‐extending fish through habitat simplification
Source: J Anim Ecol. 2026 May 11;95(8):1386–400. doi: 10.1111/1365-2656.70270 (PMC13439587; doi:10.1111/1365-2656.70270)
Supplement: Supplementary file 1 [file JANE-95-1386-s001.docx]

# Appendix S1

# ****Figure S1.** Daily sea temperature time series at the study reef (139.125° E, 34.375° N) from January 2021 to May 2024, extracted from the Marine Heatwave Tracker (marineheatwavetracker.org). The solid black line shows observed daily temperatures, with shaded areas indicating periods when temperatures exceeded the long-term seasonal baseline (marine heatwave conditions). Dashed lines represent seasonal climatologies. Vertical lines indicate periods when fish behavioural observations were conducted, coloured by marine heatwave category (black = non-heatwave, orange = moderate heatwave, red = strong heatwave).**

**(A)**

**(B)**

**(C)**

**Figure S2.** Example of flight initiation distance (FID) measurement for Pomacentrus coelestis in situ. (A) The focal fish (circled) prior to the simulated predator approach. (B) The measured distance (cm) at which the fish elicited a flight initiation response as the diver advanced the threat stimulus (metal ruler attached to a PVC frame). (C) The position of the fish immediately after initiating flight, with the red line indicating the escape direction of the fish at the point of response.

**Reef: p ≥ 0.058**

**MHW: p > 0.580**

**Figure S3. Mean (± SE) body size of *Pomacentrus coelestis* across reefs (Control, OW, OAW) and marine heatwave (MHW) periods (non-heatwave, strong). Bars show mean total length (cm) at the individual level, with reef colours (Control = sky blue, OW = gold, OAW = red) and MHW status indicated by fill pattern (striped = non-heatwave, solid = strong). Body size did not differ significantly among reefs in post hoc pairwise comparisons (all p ≥ 0.058) and was unaffected by MHW period (χ² = 0.31, p = 0.576) or the reef × MHW interaction (χ² = 0.28, p = 0.869; Appendix S1). Error bars are not shown for the Control reef during the strong heatwave, as only one individual was detected on transects during this period.**

**Table S1.** Seawater chemistry across reefs in 2022, 2023, and 2024. Mean (± SD) values of seawater temperature, salinity, pH, and calculated *p*CO_2_ (µatm) at the Control, OW, and OAW reefs during November 2022 (17–18, moderate heatwave), June 2023 (8–14, non-heatwave), July 2023 (21–27, strong heatwave), and May 2024 (19–24, non-heatwave). Salinity and pH were not sampled at the Control reef during November 2022. Seawater chemistry data were collected concurrently with fish community surveys (n = 3–4 per metric per reef). Long-term chemistry data (2011–2018) were extracted from Agostini et al. (2021). *p*CO_2_ values were calculated using in situ temperature, salinity, and pH measurements combined with total alkalinity data from Cattano et al. (2020) (OAW reef) and Agostini et al. (2021) (OW and Control reefs). Calculations were performed in CO2SYS for Excel (Pierrot et al., 2006) using constants from Mehrbach et al. (1973) refit by Dickson and Millero (1987). Long-term temperature deltas, pH and *p*CO_2_ data long-term pH and *p*CO₂ data (2011–2018) were extracted from Agostini et al. (2021), while for the OAW reef MHW = Marine Heatwave

| November 2022: Moderate Heatwave | | | | | | | | | | | | | |  |
| --- | --- | --- | --- | --- | --- | --- | --- | --- | --- | --- | --- | --- | --- | --- |
| **Reef** | | **pH (NBS)** | | | **Temperature (°C)** | | | | **Salinity (ppt)** | | | | ***p*CO_2_ (µatm)** |  |
| Control + MHW | | – | | | 20.0 (±0.1) | | | | – | | | | – |  |
| OW + MHW | | 8.30 (±0.04) | | | 23.5 (±<0.1) | | | | – | | | | – |  |
| OAW + MHW | | 7.80 (±0.04) | | | 23.9 (±<0.1) | | | | – | | | | – |  |
| June 2023: Non-Heatwave | | | | | | | | | | | | | |  |
| **Reef** | **pH (NBS)** | | | **Temperature (°C)** | | | | **Salinity (ppt)** | | | | ***p*CO_2_ (µatm)** | |  |
| Control | 8.13 (±0.01) | | | 21.1 (±0.1) | | | | 34.1 (±0.1) | | | | 447 | |  |
| OW | 8.13 (±0.04) | | | 22.03 (±0.6) | | | | 34.2 (±0.1) | | | | 455 | |  |
| OAW | 7.98 (±0.04) | | | 22.3 (±0.2) | | | | 34.3 (±0.1) | | | | 666 | |  |
| July 2023: Strong Heatwave | | | | | | | | | | | | | |  |
| **Reef** | **pH (NBS)** | | | **Temperature (°C)** | | | | **Salinity (ppt)** | | | | ***p*CO_2_ (µatm)** | |  |
| Control | 8.12 (±0.0) | | | 20.7 (±0.1) | | | | 34.0 (±0.1) | | | | 459 | |  |
| OW + MHW | 8.19 (±0.1) | | | 27.1 (±1.2) | | | | 34.4 (±0.1) | | | | 392 | |  |
| OAW + MHW | 8.00 (±0.2) | | | 27.6 (±0.5) | | | | 34.4 (±0.1) | | | | 650 | |  |
| May 2024: Non-Heatwave | | | | | | | | | | | | | |  |
| **Reef** | **pH (NBS)** | | | **Temperature (°C)** | | | | **Salinity (ppt)** | | | | ***p*CO_2_ (µatm)** | |  |
| Control | 8.24 (±0.01) | | | 18.5 (±<0.1) | | | | 30.8 (±0.8) | | | | 341 | |  |
| OW | 8.18 (±0.04) | | | 20.5 (±<0.1) | | | | 33.6 (±2.6) | | | | 388 | |  |
| OAW | 8.09 (±0.05) | | | 22.9 (±2.5) | | | | 35.7 (±0.9) | | | | 494 | |  |
| Long-term averages of studied reefs | | |  | | | |  | | |  | | | |  |
| **Reef** | **pH (NBS)** | | | | | **Temperature δ(°C)** | | | | | ***p*CO_2_ (µatm)** | | | |
| Control | 8.08 (±0.03) | | | | | – | | | | | 364 (±9) | | | |
| OW | 8.14 (±0.06) | | | | | +0.81 (±0.3) | | | | | 309 (±46) | | | |
| OAW | 7.81 (±0.09) | | | | | +0.84 (±0.3) | | | | | 768 (±225) | | | |

**Table S2.** Summary of survey periods, marine heatwave (MHW) status and characteristics, and data collected at each reef location (Control, OW = ocean warming, OAW = ocean warming and acidification). MHW categories follow Hobday et al. (2018): Category I = Moderate (+1–2 °C above climatological mean); Category II = Strong (+2–3 °C above climatological mean). MHW characteristics (mean/max intensity and cumulative intensity) are averaged across reefs. Colour shading indicates MHW status: **green** = no heatwave; **amber** = Category I (moderate); **red** = Category II (strong). * Control reef sampled 9 days prior to formal MHW onset; treated as strong heatwave conditions given anomalously elevated SST (+2.5 °C above climatological mean) at time of sampling.

| **Survey Period** | **MHW Category** | **MHW Dates** | **MHW Characteristics** | **Reefs Sampled** | **Data Collected** |
| --- | --- | --- | --- | --- | --- |
| **July 2021** | None | — | No heatwave detected during this survey period | Control, OW, OAW | Behavioural video observations (focal fish) |
| **May 2022** | Category I (Moderate) | 2 May – 10 May 2022 | Mean +1.99 °C; Max +2.16 °C; Cumulative 17.92 °C·days | Control, OW, OAW | Behavioural video observations (focal fish) |
| **August 2022** | Category I (Moderate) | 26 Jul – 12 Aug 2022 | Mean +2.11 °C; Max +2.57 °C; Cumulative 37.96 °C·days | Control, OW, OAW | Behavioural video observations (focal fish) |
| **November 2022** | Category I (Moderate) | 25 Oct – 20 Dec 2022 | **Mean:** +2.28 °C; **Max:** +2.67 °C; **Cumulative:** 130.11 °C·days | Control, OW, OAW | Behavioural video observations (focal fish) |
| **June 2023** | None | — | No heatwave detected during this survey period | OW, OAW | Behavioural video observations (focal fish) |
| **July 2023** | Category II (Strong) | Formal onset: 23 Jul 2023 Control sampled 14 Jul 2023 (9 days pre-onset) FID: 25–30 Jul 2023 | Max +3.51 °C; Cumulative 78.87 °C·days | Control, OW, OAW | Behavioural video observations (focal fish). Flight initiation distance (FID) trials (25–30 Jul). Population density surveys (UVC belt transects). Zooplankton sampling. |
| **May 2024** | None | — | No heatwave detected during this survey period | Control, OW, OAW | Behavioural video observations (focal fish). Population density surveys (UVC belt transects). Habitat height assessments |

**Table S3. Final GLMM for bite rate and resulting statistical outputs across levels of shoal size, reef location, and MHW status.** Models were fitted using generalized linear mixed models (GLMMs) with a Tweedie distribution (log link) in the glmmTMB package in R. Year was included as a random effect. Coefficients are presented as model estimates ± standard error (SE) on the log scale, alongside the Wald z statistic and p-values. χ² values are from Type III Wald χ² tests of fixed effects. Where relevant, Tukey-adjusted post hoc contrasts from the emmeans package are reported. DHARMa residual diagnostics are included to test for uniformity, dispersion, and zero inflation. Bold P-values denote significant effects (P < 0.05). df = degrees of freedom.

| **Effect / Contrast** | **Estimate ± SE** | **Test statistic** | **χ²** | **df** | **p (coefficient)** | **p (χ²)** |
| --- | --- | --- | --- | --- | --- | --- |
| Shoal size | 0.015 ± 0.003 | z = 5.27 | 27.80 | 1 | <0.001 | <0.001 |
| Location (OW) | -0.040 ± 0.087 | z = -0.46 | 0.21 | 2 | 0.644 | 0.899 |
| Location (OAW) | -0.032 ± 0.097 | z = -0.33 |  |  | 0.739 |  |
| MHW (Moderate) | 0.075 ± 0.068 | z = 1.11 | 1.54 | 2 | 0.268 | 0.463 |
| MHW (Strong) | 0.104 ± 0.118 | z = 0.89 |  |  | 0.376 |  |
| DHARMa diagnostics: No deviation from uniformity (KS p = 0.087), no overdispersion (p = 0.896). | | | | | | |

**Table S4. Final GLMM for retreat rate and resulting statistical outputs across levels of shoal size, reef location, and MHW status.** Models were fitted using generalized linear mixed models (GLMMs) with a Tweedie distribution (log link) in the glmmTMB package in R. Year was included as a random effect. Coefficients are presented as model estimates ± standard error (SE) on the log scale, alongside the Wald z statistic and p-values. χ² values are from Type III Wald χ² tests of fixed effects. Tukey-adjusted post hoc contrasts from the emmeans package are reported for MHW levels. DHARMa residual diagnostics are included to test for uniformity, dispersion, and zero inflation. Bold P-values denote significant effects (P < 0.05). df = degrees of freedom.

| **Effect / Contrast** | **Estimate ± SE** | **Test statistic** | **χ²** | **df** | **p (coefficient)** | **p (χ²)** |
| --- | --- | --- | --- | --- | --- | --- |
| Shoal size | -0.068 ± 0.025 | z = -2.67 | 7.11 | 1 | 0.008 | 0.008 |
| Location (OW) | 0.958 ± 0.545 | z = 1.76 | 4.61 | 2 | 0.079 | 0.100 |
| Location (OAW) | 0.489 ± 0.570 | z = 0.86 |  |  | 0.391 |  |
| MHW (Moderate) | -0.196 ± 0.289 | z = -0.68 | 5.37 | 2 | 0.498 | 0.068 |
| MHW (Strong) | 0.767 ± 0.405 | z = 1.90 |  |  | 0.058 |  |
| Contrast: Non-heatwave – Moderate | 0.196 ± 0.289 | z = 0.68 |  |  | 0.776 |  |
| Contrast: Non-heatwave – Strong | -0.767 ± 0.405 | z = -1.90 |  |  | 0.140 |  |
| Contrast: Moderate – Strong | -0.963 ± 0.418 | z = -2.30 |  |  | 0.055 |  |
| DHARMa diagnostics: No deviation from uniformity (KS p = 0.870), no overdispersion (p = 0.936), no zero-inflation (p = 1). | | | | | | |

**Table S5. Final GLMM for activity levels (%) and resulting statistical outputs across levels of shoal size and reef location.** Models were fitted using generalized linear mixed models (GLMMs) with a Gaussian distribution (log link) in the glmmTMB package in R. Year was included as a random effect. Coefficients are presented as model estimates ± SE on the log scale, with corresponding Wald z statistics and p-values. χ² values are from Type III Wald χ² tests of fixed effects. Tukey-adjusted post hoc contrasts from the emmeans package are reported for location. DHARMa residual diagnostics are included to test for uniformity and dispersion. Bold P-values denote significant effects (P < 0.05). df = degrees of freedom.

| **Effect / Contrast** | **Estimate ± SE** | **Test statistic** | **χ²** | **df** | **P(coefficient)** | **P (χ²)** |
| --- | --- | --- | --- | --- | --- | --- |
| Shoal size | 0.008 ± 0.003 | t = 2.94 | 8.61 | 1 | 0.003 | 0.003 |
| Location (OW) | -0.169 ± 0.079 | t = -2.13 | 6.86 | 2 | 0.033 | 0.032 |
| Location (OAW) | -0.246 ± 0.098 | t = -2.53 |  |  | 0.012 |  |
| Contrast: Control – OW | 0.169 ± 0.079 | t = 2.13 |  | 85 | 0.090 |  |
| Contrast: Control – OAW | 0.246 ± 0.098 | t = 2.53 |  | 85 | 0.036 |  |
| Contrast: OW – OAW | 0.078 ± 0.078 | t = 0.99 |  | 85 | 0.583 |  |
| DHARMa diagnostics: No deviation from uniformity (KS p = 0.125), no overdispersion (P = 0.600). | | | | | | |

**Table S6. Final GLMM for refugia use (time spent near refugia, %) and resulting statistical outputs across levels of shoal size and MHW status.** Models were fitted using generalized linear mixed models (GLMMs) with a Tweedie distribution (log link) in the glmmTMB package in R. Year was included as a random effect. Coefficients are presented as model estimates ± SE on the log scale, with Wald z statistics and p-values. χ² values are from Type III Wald χ² tests of fixed effects. Tukey-adjusted post hoc contrasts from the emmeans package are reported for MHW levels. DHARMa residual diagnostics are included to test for uniformity, dispersion, and zero inflation. Bold P-values denote significant effects (P < 0.05). df = degrees of freedom.

| **Effect / Contrast** | **Estimate ± SE** | **Test statistic** | **χ²** | **df** | **P(coefficient)** | **P (χ²)** |
| --- | --- | --- | --- | --- | --- | --- |
| Shoal size | -0.210 ± 0.048 | z = -4.40 | 19.33 | 1 | <0.001 | <0.001 |
| MHW (Moderate) | -0.622 ± 0.314 | z = -1.98 | 4.09 | 2 | 0.047 | 0.129 |
| MHW (Strong) | -0.028 ± 0.410 | z = -0.07 |  |  | 0.946 |  |
| Contrast: Non-heatwave – Moderate | 0.622 ± 0.314 | z = 1.98 |  |  | 0.116 |  |
| Contrast: Non-heatwave – Strong | 0.028 ± 0.410 | z = 0.07 |  |  | 0.997 |  |
| Contrast: Moderate – Strong | -0.594 ± 0.456 | z = -1.30 |  |  | 0.393 |  |
| DHARMa diagnostics: No deviation from uniformity (KS p = 0.700), no overdispersion (P = 0.368), no zero-inflation (p = 1). | | | | | | |

**Table S7.** Final selected model (GLM, Tweedie family with log-link) for *Pomacentrus coelestis* flight initiation distance (FID) across shoal size and reef type (Control, OW, OAW). Models presented in Table S6 are those reported in the main Results section. Bold P-values denote significant effects (P < 0.05). df = degrees of freedom.

| **Effect / Contrast** | **Estimate ± SE** | **Test statistic** | **χ²** | **df** | **P (coefficient)** | **P (χ²)** |
| --- | --- | --- | --- | --- | --- | --- |
| Intercept (Control) | 2.936 ± 0.124 | z = 23.62 | 558.02 | 1 | <0.001 *** | <0.001 |
| Shoal Size | -0.027 ± 0.013 | z = -2.14 | 4.57 | 1 | 0.033 * | 0.033 |
| Location (OW) | -0.002 ± 0.125 | z = -0.02 | 4.61 | 2 | 0.985 | 0.100 |
| Location (OAW) | 0.322 ± 0.176 | z = 1.83 |  |  | 0.067 |  |
| DHARMa diagnostics: No deviation from uniformity (KS test p = 0.862), no overdispersion (P = 0.968), no zero-inflation detected (P = 1). | | | | | | |

**Table S8. Final GLMM for shoal size and resulting statistical outputs across levels of reef location and MHW status.** Models were fitted using generalized linear mixed models (GLMMs) with a negative binomial distribution (log link) in the glmmTMB package in R. Year was included as a random effect. Coefficients are presented as model estimates ± SE on the log scale, with Wald z statistics and p-values. χ² values are from Type III Wald χ² tests of fixed effects. Tukey-adjusted post hoc contrasts from the emmeans package are reported for reef location and MHW status. DHARMa residual diagnostics are included to test for uniformity and dispersion. Bold P-values denote significant effects (P < 0.05). df = degrees of freedom.

| **Effect / Contrast** | **Estimate ± SE** | **Test statistic** | **χ²** | **df** | **P (coefficient)** | **P (χ²)** |
| --- | --- | --- | --- | --- | --- | --- |
| Location (OW) | -0.061 ± 0.275 | z = -0.22 | 28.35 | 2 | 0.824 | <0.001 |
| Location (OAW) | -1.267 ± 0.315 | z = -4.03 |  |  | <0.001 |  |
| MHW (Moderate) | 0.898 ± 0.200 | z = 4.49 | 32.13 | 2 | <0.001 | <0.001 |
| MHW (Strong) | -0.985 ± 0.482 | z = -2.05 |  |  | 0.041 |  |
| Contrast: Control – OW | 0.061 ± 0.275 | z = 0.22 |  |  | 0.973 |  |
| Contrast: Control – OAW | 1.267 ± 0.315 | z = 4.03 |  |  | <0.001 |  |
| Contrast: OW – OAW | 1.205 ± 0.237 | z = 5.10 |  |  | <0.001 |  |
| Contrast: Non-heatwave – Moderate | -0.898 ± 0.200 | z = -4.49 |  |  | <0.001 |  |
| Contrast: Non-heatwave – Strong | 0.985 ± 0.482 | z = 2.05 |  |  | 0.102 |  |
| Contrast: Moderate – Strong | 1.883 ± 0.466 | z = 4.04 |  |  | <0.001 |  |
| DHARMa diagnostics: No deviation from uniformity (KS P = 0.728), no overdispersion (P = 0.240). | | | | | | |

**Table S9.** Final GLM for *Pomacentrus coelestis* population density and resulting statistical outputs across levels of reef (Control, OW, OAW) and MHW status (Non-heatwave, Strong). Models were fitted using generalized linear models (GLMs) with a Tweedie distribution (log link) in the *glmmTMB* package in R. Coefficients are presented as model estimates ± standard error (SE) on the log scale, with associated Wald z statistics and p-values. χ² values are from Type III Wald χ² tests of fixed effects. Tukey-adjusted post hoc contrasts are reported for reef location. DHARMa residual diagnostics are included to test for uniformity and dispersion. Bold P-values denote significant effects (P < 0.05). df = degrees of freedom.

| **Effect / Contrast** | **Estimate ± SE** | **z** | **p (coefficient)** | **χ²** | **df** | **P (χ²)** |
| --- | --- | --- | --- | --- | --- | --- |
| Intercept | -0.357 ± 0.651 | -0.55 | 0.584 | 0.30 | 1 | 0.584 |
| Reef (OW) | 3.104 ± 0.681 | 4.56 | <0.001 *** | 38.27 | 2 | <0.001 *** |
| Reef (OAW) | 0.000 ± 0.921 | 0.00 | 1.000 |  |  |  |
| MHW (Strong) | -1.946 ± 1.516 | -1.28 | 0.199 | 1.65 | 1 | 0.199 |
| Reef OW × MHW Strong | 1.809 ± 1.543 | 1.17 | 0.241 | 1.61 | 2 | 0.446 |
| Reef OAW × MHW Strong | 2.197 ± 1.752 | 1.25 | 0.210 |  |  |  |
| Contrast: Control – OW | -4.01 ± 0.77 | -5.20 | <0.001 *** |  |  |  |
| Contrast: Control – OAW | -1.10 ± 0.88 | -1.25 | 0.422 |  |  |  |
| Contrast: OW – OAW | 2.91 ± 0.46 | 6.28 | <0.001 *** |  |  |  |
| DHARMa diagnostics: No deviation from uniformity (KS P = 0.183), no overdispersion (P = 0.896). | | | | | | |

# ****Table S10.**** Results of a generalized linear mixed‐effects model (GLMM) testing effects of reef (Control, OW, OAW) and marine heatwave (MHW) status (non‐heatwave, strong) on total length (TL, cm) of Pomacentrus coelestis. The model was fitted with a Gaussian error distribution and log link, with transect identity as a random intercept (n = 316 individuals, 21 transects). Shown are fixed effect estimates (± SE), Type III Wald χ² tests, Tukey‐adjusted pairwise contrasts averaged over MHW status, and DHARMa diagnostic tests for model assumptions.

| **Effect / Contrast** | **Estimate ± SE** | **z / t** | **χ²** | **df** | **P** | **Notes** |
| --- | --- | --- | --- | --- | --- | --- |
| (Intercept) | 1.466 ± 0.081 | z = 18.114 | - | - | <0.001 |  |
| Reef (OW) | 0.202 ± 0.027 | z = 7.558 | - | - | <0.001 |  |
| Reef (OAW) | 0.054 ± 0.036 | z = 1.505 | - | - | 0.132 |  |
| MHW (Strong) | 0.140 ± 0.250 | z = 0.559 | - | - | 0.576 |  |
| Reef × MHW (OW) | -0.056 ± 0.251 | z = -0.223 | - | - | 0.823 |  |
| Reef × MHW (OAW) | -0.126 ± 0.274 | z = -0.458 | - | - | 0.647 |  |
|  |  |  |  |  |  |  |
| Type III Wald tests | | | | | | |
| Reef | - | - | 84.44 | 2 | <0.001 *** |  |
| MHW | - | - | 0.31 | 1 | 0.576 |  |
| Reef × MHW | - | - | 0.28 | 2 | 0.869 |  |
|  |  |  |  |  |  |  |
| Post hoc contrasts (Tukey-adjusted) |  |  |  |  |  | averaged over MHW |
| Control – OW | -0.174 ± 0.126 | t = -1.382 | - | 308 | 0.352 |  |
| Control – OAW | 0.009 ± 0.137 | t = 0.067 | - | 308 | 0.998 |  |
| OW – OAW | 0.183 ± 0.080 | t = 2.299 | - | 308 | 0.058 |  |
|  |  |  |  |  |  |  |
| Diagnostics (DHARMa) | | | | | | |
| Uniformity (KS) | - | - | D = 0.085 | - | 0.020 |  |
| Dispersion | - | - | 1.062 | - | 0.720 |  |
| Zero inflation | - | - | NaN | - | 1.000 |  |

# Table S11. Results of Kruskal–Wallis and pairwise Wilcoxon rank-sum tests comparing mean maximum canopy height (cm) among reefs.

| **Test** | **Comparison** | **χ² / Z** | **df** | **P-value** |
| --- | --- | --- | --- | --- |
| Kruskal–Wallis | — | 49.19 | 2 | < 0.001 |
| Wilcoxon post hoc (Holm-corrected) | Control vs OW | — | — | 0.022 |
|  | Control vs OAW | — | — | < 0.001 |
|  | OW vs OAW | — | — | < 0.001 |

# Table S12. Results of Kruskal–Wallis and pairwise Wilcoxon rank-sum tests comparing mean zooplankton density (individuals. m^-2^) among reefs.

| **Test** | **Comparison** | **χ² / Z** | **df** | **P-value** |
| --- | --- | --- | --- | --- |
| Kruskal–Wallis | — | 27.73 | 2 | < 0.001 |
| Wilcoxon post hoc (Holm-corrected) | Control vs OW | — | — | < 0.001 |
|  | Control vs OAW | — | — | < 0.001 |
|  | OW vs OAW | — | — | 0.025 |
